# Tooth Migration in a Female Patient with Hyperdontia: 11-Year Follow-Up Case Report

**DOI:** 10.3390/jcm12093206

**Published:** 2023-04-29

**Authors:** Agnieszka Bogdanowicz, Kaja Szwarczyńska, Sonia Barbara Zaleska, Tomasz Kulczyk, Barbara Biedziak

**Affiliations:** 1 Department of Orthodontics and Craniofacial Anomalies, Poznan University of Medical Sciences, 60-812 Poznan, Poland; 2 Orthodontic Clinic, The University Center for Dentistry and Specialized Medicine, 60-812 Poznan, Poland; 3 Department of Diagnostics, Poznan University of Medical Sciences, 60-812 Poznan, Poland

**Keywords:** hyperdontia, migration, supernumerary teeth, supplemental teeth, non-syndrome multiple supernumerary teeth

## Abstract

We described an 11-year follow-up of a patient with a non-syndrome multiple supernumerary teeth who had one extra tooth in the maxilla and four additional premolars in the mandible. Together with an additional distal migration of the second lower right premolar to the ramus of the mandible that also occurred, it comprises a unique combination of conditions that were not previously presented in the literature. We showed the significance of routine X-rays in cases of hyperdontia since the additional teeth may develop later than expected and the patient may not experience any symptoms.

## 1. Introduction

The objective of this article is to show an 11-year follow-up of an uncommon case of distinctive conditions coexisting and delayed additional teeth development.

Tooth development is a very complex process involving several stages. It starts with a dental epithelium’s thickening that forms the dental lamina. In the area of this thickened tissue, cells begin to proliferate and form the intended structures known as the placodes. Their further invagination gives rise to the later stages of tooth development known as bud, cap and bell. Meanwhile, the interchange of chemical signals between the mesenchyme and epithelium causes the mesenchyme to transform into odontoblasts, which are dentine precursors, while from the epithelium, the source of the enamel part of the tooth emerges as ameloblasts [1].

Teeth develop within the jaws and their movement through bone from their place of origin to their functional positions within the oral cavity is called tooth eruption. Eruption of second set of dentition also accomplishes root resorption and exfoliation of the first.

Teeth erupt as an outcome of the dedicated molecules’ interaction within the alveolar bone, including its resorption and formation. Most prominent theories involving this mechanism emphasize the importance of, among others, dental follicle, root elongation, alveolar bone remodeling and periodontal ligament [2,3].

During the long-lasting process of dental development, many abnormalities may occur. Both the deciduous and permanent teeth can be affected by changes in their number, morphology, location and eruption time. One of the disorders concerning the first of these types is hyperdontia. Supernumerary teeth are defined as extra teeth or tooth-like structures occurring in addition to the physiological number of 20 primary and 32 permanent teeth. The supernumerary teeth can be either erupted or remain retained [4,5,6]. It is important to specify any excess teeth in specific groups of teeth because extra teeth may coexist in combination with dental agenesis and, in those cases, the total number of teeth may stay correct despite the presence of an abnormality [7].

The prevalence of hyperdontia varies from 0.04% to 2.29% and is less common in females than in males. It is most frequent in the anterior part of the dental arch in the maxilla [5]. In permanent dentition, the affected area is usually located palatially, while in deciduous dentition, it is more often within the dental arch [8]. Furthermore, the hyperdontia typically involves the occurrence of only a single additional tooth; however, cases of many such teeth can be observed.

The classification of hyperdontia is based on the number, morphology and location of the extra teeth. In deciduous dentition, the morphology of the teeth is usually regular or conical. However, in permanent dentition, a variety of forms can be observed [9]. Classically, the irregularity of the number of teeth was divided into supplemental–identical morphology, which function as the adjacent teeth, and supernumerary–atypically shaped, whose function varies from the ones in the adjacent region [10]. The most common extra tooth is mesiodens; nevertheless, extra central and lateral incisors, extra premolars and molars, as well as paramolars and retromolars in rudimentary forms, can also be found [9].

Multiple supernumerary teeth are usually connected to syndromes such as Gardner’s syndrome, Fabry Anderson syndrome, Incontinentia Pigmenti, Familial Adenomatous Polyposis, Rubinstein–Taybi Syndrome, Nance–Horan Syndrome, Opitz G/BBB Syndrome, Oculofaciocardiodental Syndrome, Robinow Syndrome and Trichorhinophalengeal Syndrome, Kreiborg–Pakistani Syndrome, insulin-resistant diabetes mellitus with Acanthosisnigricans and developmental disorders such as cleft lip and palate, chondroectodermal dystosis and cleidocranial dysostosis [6,11,12,13].

The migration of a tooth is defined as the movement of a tooth or a tooth germ within the bone which is caused by the fact that its eruption is prevented [14,15]. The etiology of this anomaly is still unclear. There are suggestions that the migration can be caused by a cystic lesion or an odontoma [16]. In the general population, the second lower premolar migration occurs in 0.2% of the cases and is less common in males than in females (1:1.17 ratio) [17]. Due to the teeth tendency for mesial movement caused by the natural masticatory effect, the distal migration is yet to be fully examined [14].

## 2. Case Report

A 9-year-old female patient was referred to our clinic for an investigation of an unerupted tooth 11. The past medical and family history was noncontributory. The patient did not have any systemic diseases or abnormalities in the skeletal structure and has not had any tooth extractions performed. The patient’s mother has not reported any abnormalities in the development of dentition. Within the family’s medical history, no one presented with similar symptoms.

Intraoral examination revealed mixed dentition and a retained tooth 51. The occlusion was age-appropriate and no other abnormalities were detected.

**Figure 1:**
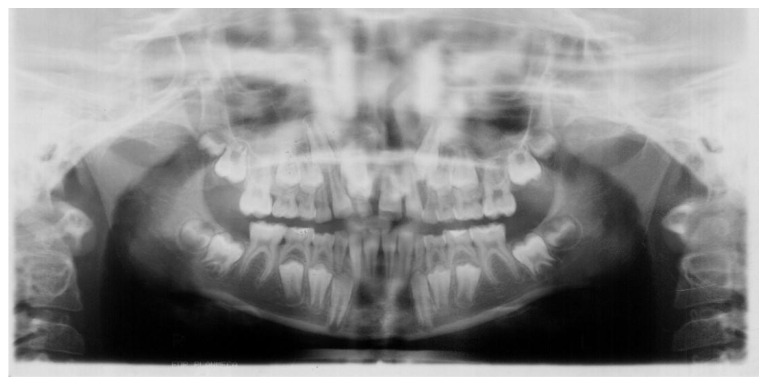
First panoramic radiograph, taken when patient was 9 years old, showing presence of all permanent teeth including third molars and one extra tooth. This supernumerary tooth was located between the cervices of the teeth 12 and 21, hindering eruption and development of the tooth 11. The distal angulation of both second lower premolars was also noticed. The alpha and beta angles of the tooth 13 are 16° and 3°.

First, a panoramic radiograph (Figure 1) showed a full permanent dentition, including third molars and one extra tooth. This supernumerary tooth was located between the cervices of the teeth 12 and 21, hindering the eruption and development of the tooth 11. The distal angulation of both second lower premolars was also noticeable. The angle alpha, which is measured between the long axis of the impacted canine and the upper midline, is 16°. The angle beta, which lies between the long axis of the impacted canine and the long axis of the adjacent lateral incisor, is 3°.

The decision was made to extract the extra tooth in order to enable the eruption of the impacted 11.

**Figure 2:**
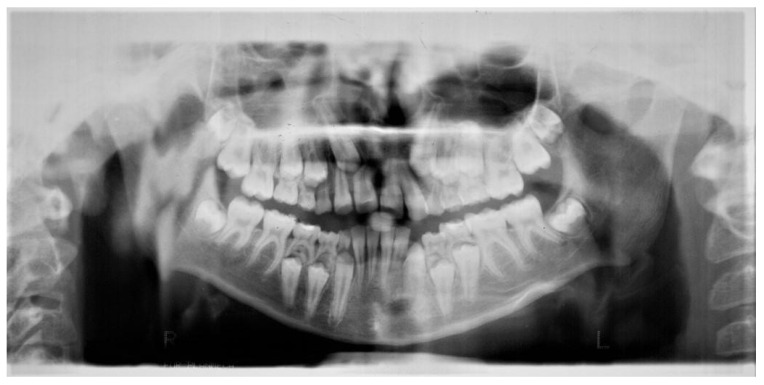
Panoramic radiograph taken two years later, when patient was 11 years old, showing the germ of the additional tooth located above the germ of the tooth 34 and crypts of the developing additional teeth located above the germs of the teeth 44 and 45. Additionally, the incorrect eruption path of the tooth 13 is visible. Unlike the tooth 45, which increases its distal inclination, the germ of the tooth 35 resorbs the distal root of the tooth 75.

A second panoramic radiograph (Figure 2) taken after two years, when the patient was 11 years old, which showed a germ of the next additional tooth above the germ of the tooth 34 and crypts of the developing extra teeth located above teeth 44 and 45.

The tooth 11 resorption was noticed, in addition to an irregular eruption path of the tooth 13, the increase in a distal inclination of the tooth 45 and the resorption of the distal root of the tooth 75 by the germ of the 35. Alpha and beta angles of the tooth 13 increased to 25° and 23°.

**Figure 3:**
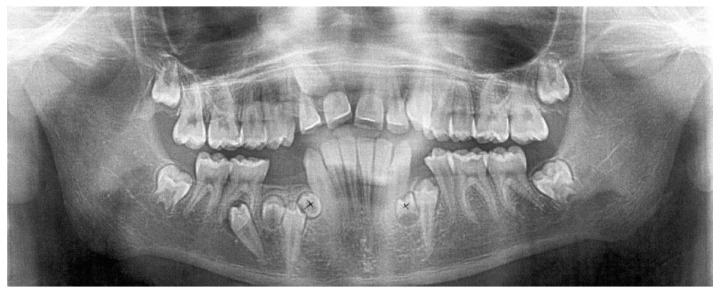
Panoramic radiograph, taken one year later, when patient was 12 years old, showing three additional mandibular premolars: the first one located between the apex of the tooth 33 and the crown of the impacted tooth 34; the second one, found between the apex of the root of the tooth 43 and the crown of the impacted tooth 44; and the third one, found between the mesial root of the tooth 46 and the crown of the impacted tooth 44. The distal migration of the tooth 45 was noticeable and its crown was located in the projection of the roots of the tooth 46. The tooth 13 was located between 1/3 of the cervical part of the root of the tooth 12 and the tooth 11. Alpha and beta angles of the tooth 13 increased to 29° and 20°.

The next X-ray picture (Figure 3) was taken after one year of follow-up when the patient was 12 years old and already had three additional mandibular premolars: the first one located between the apex of the tooth 33 and the crown of the impacted tooth 34; the second one, found between the apex of the root of the tooth 43 and the crown of the impacted tooth 44; and the third one, between the mesial root of the tooth 46 and the crown of the impacted tooth 44. The distal migration of the tooth 45 was noticeable and its crown was located in the projection of the roots of the tooth 46. The tooth 13 was located between 1/3 of the cervical part of the root of the tooth 12 and the tooth 11. The alpha angle of the tooth 13 increased to 29° and the beta angle decreased to 20°.

At this stage, the patient was referred to a dental surgeon to extract the first and the second additional teeth.

**Figure 4:**
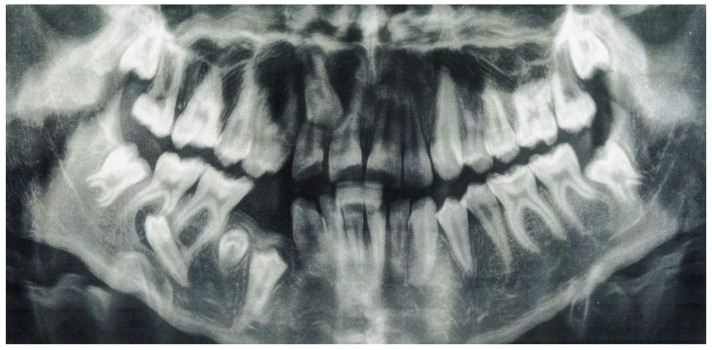
Panoramic radiograph, taken one year later, when patient was 13 years old, showing the germ of the third additional tooth located in the standard position of the 45 tooth—between the crown of the impacted tooth 44 and the mesial root of the tooth 46. The further migration of the tooth 45 is visible—the crown can be seen in the projection of the apex of the mesial root of the tooth 47. Furthermore the impacted tooth 13, located between 1/3 cervical part of the roots of the teeth 12 and 11, is also visible.

The next panoramic radiograph (Figure 4), taken after one year when the patient was 13 years old, showed the germ of the third additional tooth located in the standard position of the tooth 45—between the crown of the impacted tooth 44 and the mesial root of the tooth 46. The further migration of the tooth 45 is visible—the crown can be seen in the projection of the apex of the mesial root of the tooth 47. Furthermore, the impacted tooth 13, located between 1/3 cervical part of the roots of the teeth 12 and 11, is also visible.

**Figure 5:**
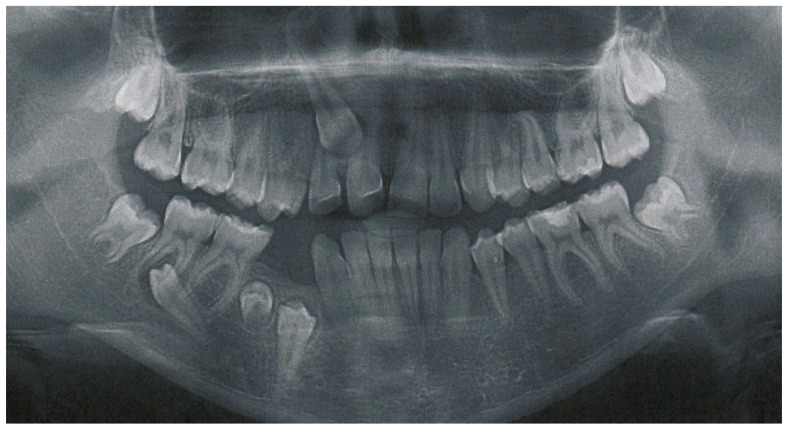
Panoramic radiograph, taken two months later, showing the continued distal migration of the tooth 45, here located between the roots of the tooth 47.

The next X-ray picture (Figure 5), taken two months later, revealed even further distal migration of the tooth 45, which was then located between the roots of the tooth 47. Alpha and beta angles of the tooth 13 are both 27° at this point. The decision was made to extract the impacted tooth 13.

**Figure 6:**
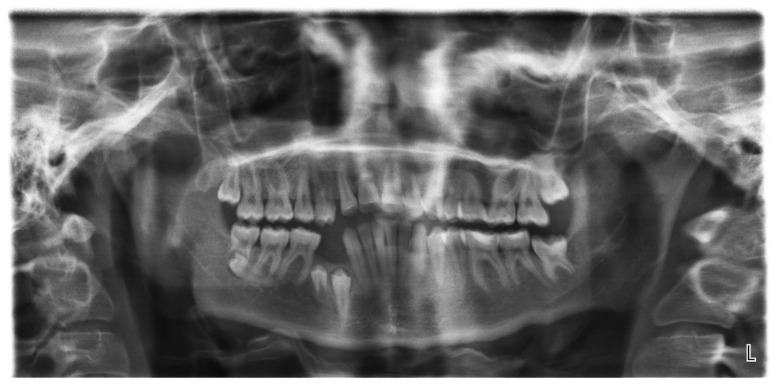
Panoramic radiograph, taken two years later, when patient was 15 years old, showing the further distal migration of the tooth 45 above the mandibular canal (its crown can be seen to overlap the roots of the tooth 48), as well as the still unerupted impacted tooth 44 and the third additional tooth.

Two years later, when the patient was 15 years old, another panoramic radiograph was taken (Figure 6), which showed continued distal migration of the tooth 45 above the mandibular canal (its crown can be seen to overlap the roots of the tooth 48), as well as the still unerupted impacted tooth 44 and the third additional tooth.

**Figure 7:**
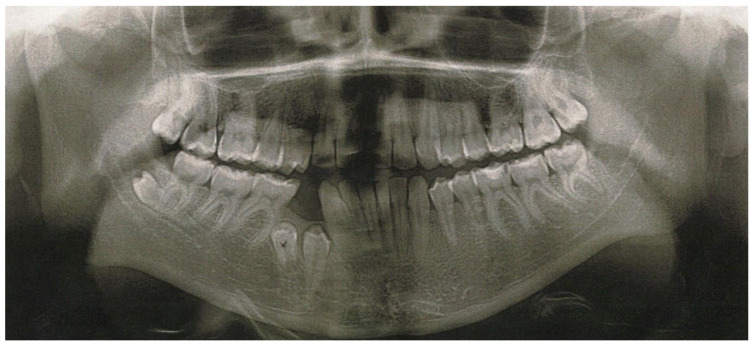
Panoramic radiograph, taken one year later, when patient was 16 years old, showing the further migration of the tooth 45—the germ was then located above the mandibular canal, distally from the roots of the tooth 48. The impacted tooth 44 and the third additional tooth had not changed their position since the previous X-ray picture had been taken. Furthermore, the crypt of the forming fourth additional tooth in the projection of 1/3 middle part of the root of the tooth 35 appeared.

One year later, the X-ray picture (Figure 7) revealed the further migration of the tooth 45—the germ was then located above the mandibular canal, distally from the roots of the tooth 48. The impacted tooth 44 and the third additional tooth had not changed their position since the previous X-ray picture had been taken. Furthermore, the crypt of the forming fourth additional tooth in the projection of 1/3 middle part of the root of the tooth 35 appeared. Thus, the decision to extract the third additional tooth was made.

**Figure 8:**
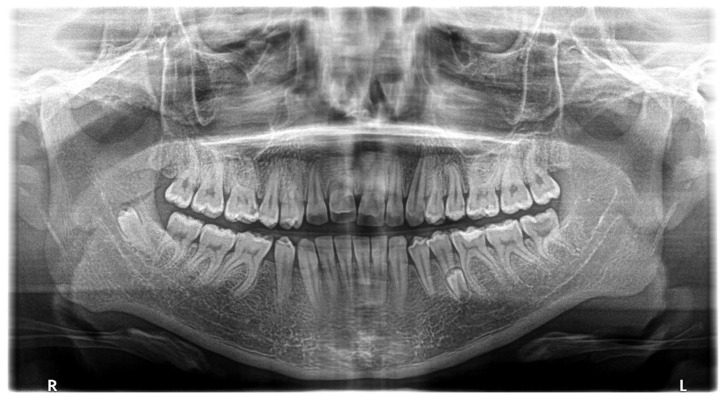
Panoramic radiograph, taken four years later, when the patient was 20 years old, showing the germ of the tooth 45, which was found in the ramus of the mandible above the mandibular canal at the same height as the crowns of the lower teeth. The fourth additional tooth had developed and located itself in the projection of 1/3 of the apical part of the root of the tooth 35. Additionally, a resorption of the root of the tooth 11 became noticeable.

Four years later, when the patient was 20 years old (Figure 8), the tooth 45 was found in the ramus of the mandible above the mandibular canal at the same height as the crowns of the lower teeth. The fourth additional tooth had developed and located itself in the projection of 1/3 of the apical part of the root of the tooth 35. Additionally, a resorption of the root of the tooth 11 became noticeable.

The patient stayed under a long, complicated, multi-specialty care for many years. All the decisions regarding treatment and times to undergo dental extractions were thoroughly discussed between an orthodontist, a dental surgeon and a pedodontist. The crucial aspects considered were the stage of development of the child, her age, her ability to cooperate, number and position of the supernumerary teeth, but also the development and proximity of adjacent anatomical structures. In this case, the decision was made to postpone the dental surgery as long as possible since the risk of damaging still developing adjacent anatomical structures and the likelihood of lack of cooperation and trauma for the smaller child was greater; thus, the risks simply outweighed the possible benefits.

## 3. Discussion

The etiology of supernumerary teeth is still unclear; however, there are a number of theories explaining the origins of this anomaly. The localized and independent hyperactivity of the dental lamina is the most accepted cause for the development of supernumerary teeth [6]. Other theories include the following: the theory of atavism as a dental pattern of protoplasts reinstatement; the theory of dichotomy of the tooth germ, which suggests the formation of the supernumerary teeth by the extra division of the dental lamina.; the vascular theory where the sphenopalatine artery does not atrophy.; the genetic theory, which proves that this phenomenon occurs more frequently amongst members of the same family; and the theory of the organogenesis disorders, where the dysfunction of the visceral arch during the embryonic development leads to supernumerary teeth germs development [5,9]. As mentioned in the Introduction, multiple supernumerary teeth are usually connected to syndromes, and non-syndrome multiple extra teeth occur less frequently—in less than 1% of the cases and are more prevalent in males than females [6,11,12,13,18]. According to the literature, 76–86% of all the cases of hyperdontia are cases of a single extra tooth and 12–23% are double supernumerary, whereas less than 1% are cases of multiple supernumerary teeth [19]. In non-syndromic multiple supernumerary teeth, the extra teeth are most often found in the lower premolars’ region (8–9.1%) [20].

Due to the reasons mentioned in the literature, only a few cases of multiple supernumerary teeth without any associated syndromes can be found. Akgun et al. described the case of a non-syndrome patient with supernumerary teeth that occurred with an interval of several years [6]. Türkkahraman et al. described a non-syndrome case with bilateral supplemental maxillary canines [21]. Najack et al. [22] presented a report of two cases with multiple supernumerary teeth without any syndrome and emphasized the advice of performing routine orthopantomograms in patients with hyperdontia.

It should be noted that hyperdontia in the primary dentition may suggest a similar anomaly in the permanent dentition [23] Hyperdontia may appear in the late periods of odontogenesis as supernumerary teeth can develop at any age; however, in 95% of the cases, they are diagnosed between the ages of 5 and 25 [24]. Research by Komorowska and Drelich revealed that in 49.5% of the cases, the development of supernumerary teeth was consistent with the development of typical teeth, delayed in 39.3%, and accelerated in 11.3% of the examined patients [25].

The information in the literature overview about impacted teeth migration is scarce and the etiology of this anomaly is still unclear. Distinction between canine and premolar migration was observed by Peck (1998) during the investigation of cases of bilateral migration—the former seemed to have a genetic foundation while the latter casual or idiopathic [14].

In the presented case, the impacted second premolar migrated to the mandibular ramus, unlike the cases reported by Ackuaku and Sharma [15], who reported two incidents where impacted premolars were positioned deep into the mesial root of the lower first permanent molar. Mortazavi et al. [26], Infante-Cossio et al. [22] and Shahoon and Esmaeili [20] reported premolar migration to the mandibular angle. Okada et al. [27] reported movement to the mandibular condyle and coronoid process. Fuziy et al. [28] described migration to the mandibular condyle, while Orton and McDonald [29] reported movement to the coronoid process. Alves et al. [14] described the mandibular second premolar that was positioned in the mandibular notch area.

It has been proven that the second lower premolar’s migration occurs more frequently when its germ is in distal inclination during the development and when the first lower molar is prematurely extracted, which increases the chance of migration to 5–10% [14,30]. In that case, the direction of the crown sets the alignment of the movement of the second premolar during the distal migration. When it reaches the roots of the second molar, it rotates towards the position of occlusion [31]. Usually, in this case, it erupts mesially to the second lower molar; however, in the literature overview, some cases of its migration up to the coronoid process can be found and women are more prone to this condition [16].

In most cases, supernumerary teeth fail to erupt and remain impacted. Supernumerary premolars are at 75% risk of not erupting [20]. Supernumerary teeth may also cause complications such as delayed eruption of permanent teeth, rotation or displacement of adjacent teeth, dilaceration, root resorption, impaction, fistulas and cystic formation [11,32]. It is important to diagnose supernumerary teeth as early as possible to avoid or minimize complications. Extra teeth in most cases are non-symptomatic and are diagnosed accidentally through routine X-rays preceding orthodontic treatment [33].

The treatment method in case of hyperdontia depends on many systemic factors, such as the patient’s health, age, possible cooperation, their choice regarding the method of treatment and local factors which include the location of the additional/supernumerary tooth, stage of its formation, proximity of the adjacent anatomical structures and the occlusion [6,9,34].

In case of deciduous teeth, 75% of the patients with hyperdontia suffer no additional complications, as those teeth erupt in the dental arch. However, they should remain under observation since there is a risk of supernumerary teeth occurring in the permanent dentition (20–50%) [9].

In permanent dentition, there are two methods of treatment-conservative and surgical management.

The former should be considered in case of asymptomatic impacted teeth, when there are no resorptions, no adjacent teeth’s displacement, no formation of the pathological structures such as cysts or odontomas or when the risk of extracting it surpasses the treatment benefits. If such an approach is selected the necessity of control X-ray examinations and vigilance towards detecting any potential pathological conditions associated with hyperdontia emerges.

There are, however, a number of circumstances when the surgical approach is preferred: delayed or impeded development of permanent teeth, tooth displacement by the additional or supernumerary one, other related pathological changes or the possible negative impact of the additional or supernumerary tooth on other ongoing dental treatment [34]. In those cases, extraction or orthodontic surgical procedure involving pulling the tooth into the arch should be considered [15]. There is no widely accepted consensus on the exact moment of undertaking such surgical interventions. There are papers in the literature that support the prompt extraction of the additional/supernumerary teeth, especially the ones in the frontal part of the dental maxillary arch, in order to utilize the eruption’s potential of the impacted teeth and avoid further complications caused by hyperdontia [35,36,37]. Moreover, as the vertical development of the incisive bone progresses, the difficulty of the extraction procedure increases. It is especially prominent in the case of inverted teeth and their germs. Other authors [36,38] point out that there are more benefits when the extraction of the additional or supernumerary tooth is carried out after the development of the roots of the adjacent teeth is complete. It decreases the risk of negative influence the procedure can have on them. The significance of better cooperation of more mature patients cannot be neglected.

Regardless of the preferred approach, the physician has to thoroughly diagnose the patient, carefully consider and choose all the treatment options, and obtain the patient’s consent to the proposed therapy.

## 4. Conclusions

In summary, teeth development is a complicated process influenced by various factors and regulated on many levels, which can take place over different time periods. As proven in this case report, supernumerary teeth may appear in the late periods of odontogenesis. In cases of hyperdontia, the follow-up period and routine X-ray examinations are very important for treatment. It is essential for patients with hyperdontia to remain under medical supervision and this case proves how important it is to observe teeth development by re-taking X-rays of these patients every couple of years. 

An intraosseous distal migration of the mandibular left second premolar is an extremely rare condition.

The presence of an intraosseous distal migration does not seem to be directly connected with non-syndrome multiple supernumerary teeth.

There are many hypotheses explaining hyperdontia and dental migration; however, the direct cause remains unclear to us.

## Data Availability

No new data were created or analyzed in this study. Data sharing is not applicable to this article.
